# Colour Calibration of a Head Mounted Display for Colour Vision Research Using Virtual Reality

**DOI:** 10.1007/s42979-021-00855-7

**Published:** 2021-10-27

**Authors:** Raquel Gil Rodríguez, Florian Bayer, Matteo Toscani, Dar’ya Guarnera, Giuseppe Claudio Guarnera, Karl R. Gegenfurtner

**Affiliations:** 1 Department of Psychology, Justus-Liebig-Universität Giessen, Giessen, Germany; 2 Department of Computer Science, Norwegian University of Science and Technology, Gjøvik, Norway; 3 University of York, York, UK

**Keywords:** Head mounted display, Virtual reality, Colour calibration, Colorimeter, Spectroradiometer, Colour vision, Colour constancy

## Abstract

Virtual reality (VR) technology offers vision researchers the opportunity to conduct immersive studies in simulated real-world scenes. However, an accurate colour calibration of the VR head mounted display (HMD), both in terms of luminance and chromaticity, is required to precisely control the presented stimuli. Such a calibration presents significant new challenges, for example, due to the large field of view of the HMD, or the software implementation used for scene rendering, which might alter the colour appearance of objects. Here, we propose a framework for calibrating an HMD using an imaging colorimeter, the I29 (Radiant Vision Systems, Redmond, WA, USA). We examine two scenarios, both with and without using a rendering software for visualisation. In addition, we present a colour constancy experiment design for VR through a gaming engine software, Unreal Engine 4. The colours of the objects of study are chosen according to the previously defined calibration. Results show a high-colour constancy performance among participants, in agreement with recent studies performed on real-world scenarios. Our studies show that our methodology allows us to control and measure the colours presented in the HMD, effectively enabling the use of VR technology for colour vision research.

## Introduction

Colour represents an important feature of our visual perception. We are able to recognise and segment objects easily, based on their colour [32]. In particular, we perceive the colours of objects as relatively stable despite large changes in the illuminating lights. This ability of the human visual system is known as colour constancy [14]. Colour constancy can be quantified with an index in the range 0–100$\%$, where $0\%$ means that the appearance of an object changes as much as the light reflected into the eye changes with the illumination, and $100\%$ indicates no change in the object appearance under illuminant changes.

The vast body of literature on colour constancy (e.g., Arend and Reeves [2], Kraft and Brainard [18], Hansen et al. [17], Radonjić et al. [25]) shows a large degree of variability in constancy performance, which ranges from 20 to $90\%$, depending on experimental conditions. Colour constancy seems to increase with the level of complexity of the visual scene: $30\%$ [2], in experiments with flat coloured patches, to about $90\%$ with realistic viewing conditions and stimuli [18, 20], and above $90\%$ in real-world scenarios [15]. This suggests that realistic scenes include the cues which are actually used by the visual system for discounting the contribution of the illumination. Hence, for revealing the mechanism involved in colour constancy it is crucial to conduct experiments with rich and realistic stimuli.

However, there are limitations with respect to stimulus control in the real world. Some experimental conditions are very difficult and time consuming to achieve, for example when objects or parts of the scene have to be replaced between experimental trials [17, 18]. Other manipulations are simply impossible in the real world, for example changing the physics of light to exclude mutual illumination, but see Bloj et al. [4].

In recent years, virtual reality (VR) technology has improved significantly, allowing for photorealistic real-time physical renderings, higher spatial resolution and broader field of view of the head mounted display (HMD) [1]. While VR plays a significant role in the gaming industry, lately its popularity has increased also in psychology, as a tool to conduct perceptual [9, 11, 26] or social behavioural experiments [29, 33], as well as neuroscience research [5, 31]. In fact, the available technology allows us to create immersive environments aimed at simulating the real world, and more importantly, it offers the capability to accurately control the presented stimuli. Therefore, such a technology opens a new path in overcoming the limitations of colour constancy experiments in the real world.

Nevertheless, colour vision experiments are challenging, since a calibration of the display is essential [8], i.e., a mapping between the input values versus the stimuli presented on the HMD. Recent studies proposed a framework for colour HMD calibration. In Clausen et al. [10], the authors proposed a display model to characterise two different HMDs. The work of Díaz-Barrancas et al. [13] presented as well a characterisation of two HMDs models, in their case, for a colour management system.

In this paper, we present a colour calibration of an HMD set (HTC Vive Pro Eye) using an imaging colorimeter, that acquires chromaticity and luminance values of each pixel on the screen. Thus, it will enable measuring the complexity of the realistic simulations, for instance how light is reflected from the surface of different objects and how it interacts with other objects in the scene: Lambertian and non-Lambertian bidirectional reflectance distribution functions (BRDF), and mutual reflections. The calibration is performed by considering:

1. the HMD as standard display controlled via Psychtoolbox3 [6], and
2. the HMD as a VR 3D computer-generated imagery display, controlled via Unreal Engine 4.23.

We test our calibration in a colour constancy experiment [16], and we show colour constancy indices comparable to previous works focused on real scenes. To the best of our knowledge, this is the first work on characterising the colours of a VR HMD, and its validation in a colour constancy experiment. It represents an extension of our previous work [30] in which we characterised the spectral properties of an HMD by means of a spectroradiometer.

## Methods

In this section, we present the setup and the experiments we conducted to colour calibrate the HMD using the imaging colorimeter, along with the colorimetric measurements of the stimuli we used for an immersive colour constancy experiment.

The very first step in the colour calibration of the HMD, before image acquisition, is the definition of the settings for the colorimeter; subsequently, its accuracy with respect to a reference instrument needs to be verified. In our case, such a reference is provided by a CS-2000A spectroradiometer (Konica Minolta, Chiyoda, Tokio, Japan), henceforth referred to as CS2000.

The colour constancy experiment was conducted using Unreal Engine 4.23, a real-time gaming engine. We use the CS2000 to characterise the relation between the colour specification that is provided to the engine (reflectances) and the values of chromaticity and luminance measured by the reference instrument. This characterisation allows us to verify the measurements taken with the colorimeter afterwards.

### Apparatus

**Fig. 1:**
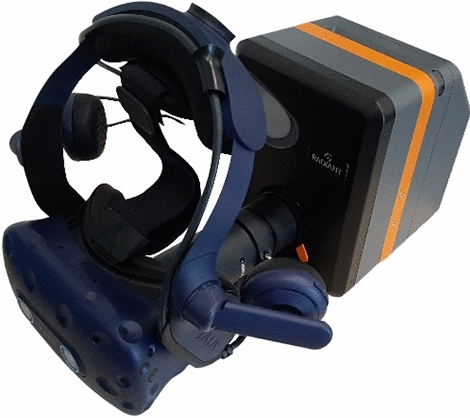
HMD colorimeter calibration setup. Vive Pro Eye and I29 with AR/VR lens.

The measurements were conducted using a desktop computer with Windows 10 as the operating system and the following specifications: Intel(R) Core(TM)i9-9900k at 3.60GHz processor, 128GB RAM, and Nvidia Titan RTX graphics card. The setup consists of an imaging colorimeter I29 (Radiant Vision Systems, Redmond, WA, USA) [24] with an AR/VR lens [23] for measuring the values presented in a Vive Pro Eye headset (HTC Corporation, Taoyuan, Taiwan), as shown in Fig. 1.

### HMD HTC Vive Pro Eye

The HMD contains two AMOLED displays of $1440 \times 1600$ pixel resolution per-eye. The frame rate is 90Hz and it has a field of view of 110$^{\circ }$. Besides, there is an embedded eye-tracking system for each eye. The HTC Vive Pro Eye headset uses Fresnel lenses, i.e., a lightweight single lens system, which is standard for HMDs to reduce weight and increase comfort. These lenses provide a wide field of view for the best immersive experience, but also cause optical distortion, particularly pincushion distortion, and chromatic aberration that is mainly visible as colour fringes at high-contrast edges, especially in the periphery. Thus, before projecting any image or 3D scene into the headset, such issues must be accounted for. In general, the technology available for creating VR projects, including rendering software, provides built-in correction for pincushion and chromatic aberration. Therefore, explicit correction for distortions is only required when no software is involved between the VR project and the headset, e.g., when the HMD is used in lieu of a normal monitor to display images generated via PsychToolbox [6]. Please notice that the issue of chromatic aberration is not completely fixed by any software. It would be preferable to solve the problem optically, but this would require a fundamental change in the optical design of the HMD by replacing the Fresnel lens with an achromatic lens (i.e., a multi-lens system), and adapting the existing software correction of the lens artefacts. We consider it a minor problem, since it affects primarily the periphery, and it has a relatively high spatial frequency that cannot be well perceived, and because it is less easily recognisable due to the typically smooth brightness transitions in naturalistic textured stimuli as used in our experiment.

In our case, for the colour calibration of the HMD, we consider both scenarios: HMD controlled via PsychToolbox, and HMD controlled via Unreal Engine and SteamVR. In the colour constancy experiment, only the latter option is used.

### I29 Imaging Colorimeter

**Fig. 2:**
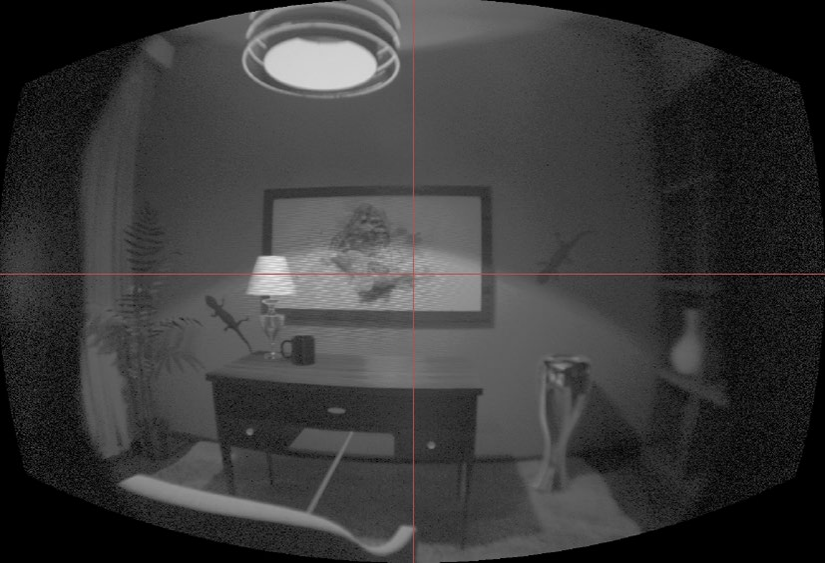
Luminance channel of an image captured by I29 from HTC Vive Pro Eye, using ProMetric.

The imaging colorimeter I29 provides measurements of chromaticity CIE (*x*, *y*) and luminance values *Y* (cd/m$^2$) with a resolution of $6576 \times 4384$ pixel and an accuracy of ± 0.003 in x and y, see Fig. 2. It is one of the currently most sophisticated imaging colorimeters available on the market. The AR/VR lens is specifically designed to measure HMDs. It provides a field of view of $120^{\circ } \times 80^{\circ }$ and allows us to position the entrance pupil at the human eye position in the HMD. The I29 must be calibrated to the HMD’s primaries first. For this purpose, we used the ProMetric version 10.11.67 [22].

In particular, we performed a *four-colour calibration* (4CC),^1^ which takes the chromaticity *xy* and luminance values of the display’s primaries and white point as input both from the I29 and from the CS2000, the latter as ground truth. Please refer to ProMetric [22] for more details on the 4CC. Note that our current calibration relies on the previous work presented in Toscani et al. [30].

To ensure that both instruments measure exactly the same location on the screen, we present a pattern beforehand and adjust them accordingly. The pattern varies intensity sinusoidally as a function of distance from the screen centre, which resembles concentric circles. The measurement aperture of the CS2000 and the measurement area of the I29 are both aligned to the centre point of this pattern. Thus, the area used for the 4CC is a circular $1^{\circ }$ area around the screen centre. We adjusted the exposure times of both instruments to be in sync with the temporal frequency of the AMOLED display (90Hz); note that we only characterise one of the two AMOLEDs, assuming they have identical characteristics.

At this stage, the primary values can be provided to the 4CC. Henceforth, all the I29 measurements are corrected based on the 4CC by the ProMetric libraries, along with other camera settings: lens distortion, colour correction options, exposure times, luminance units, plot type, etc. The 4CC was performed manually using the ProMetric GUI. For all other measurements, the I29 was controlled by Matlab via the ProMetric .NET libraries, which allowed for fully automated measurements triggered by either PsychToolbox-3 or the Unreal Engine 4.

## HMD Colour Calibration

As mentioned in the previous section, we characterise the HMD using the imaging colorimeter, in turn considering the headset as a standard display and as a 3D computer-generated imagery VR display.

### HMD Controlled via Psychtoolbox-3

The setup calibration explained in the previous section is verified by measuring the primaries directly with the colorimeter. To this purpose, we present a homogeneous image with only one of the primary colours at the time (red, green, blue), and white, using Matlab and Psychotoolbox-3 [6] to systematically change the bit value of a full-screen image. Note that we do not account for any lens distortion or aberration, since only a spatially uniform image is shown into the HMD. The colorimeter is also controlled directly via the Matlab API.

**Fig. 3:**
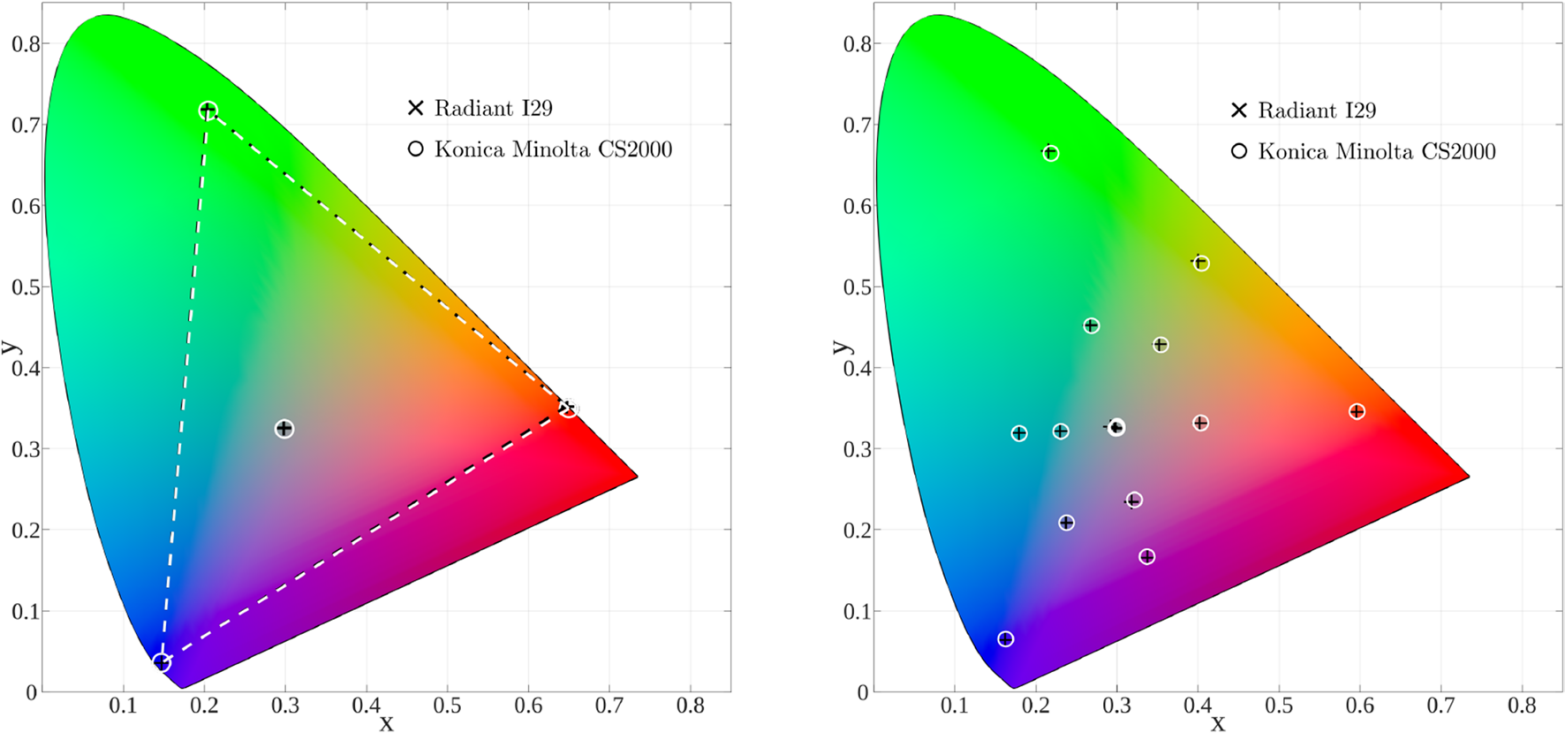
On the left: HMD primaries measured with the spectroradiometer (white dashed line) and the measured primaries using the colorimeter (black dashed line). On the right: a mixture of colours measured with the spectroradiometer (white circles) and the colorimeter (black crosses). Represented both in *xy* chromaticity diagram

Figure 3 (left) shows the chromaticity values of the primaries of the HMD measured with the reference instrument CS2000 (white rings) and I29 (black crosses). In both measurements, the HMD was controlled via PsychToolbox-3.

#### 4CC Validation

To test the 4CC, we present 16 colours on the HMD spanning the HMD gamut, and then verify that the values measured by the I29 correspond to the ones captured by the reference instrument. Specifically, we use *rgb*-triplets corresponding to the corners of a larger and a smaller cube within the *RGB* colour space, ranging from 0.2 to 0.8 and from 0.4 to 0.6, respectively. In Fig. 3 (right), we present the measurements of these 16 colours from the CS2000 (white circles) and the I29 (black crosses). Notice that both set of points are quite close in the chromaticity diagram, thus validating the current four-colour calibration.

### HMD Controlled via Unreal Engine

In this section, the HMD is controlled by Unreal Engine 4.23. We aim to control the RGB values (reflectances) introduced into the engine, and the output values measured with the colorimeter (stimuli).

**Fig. 4:**
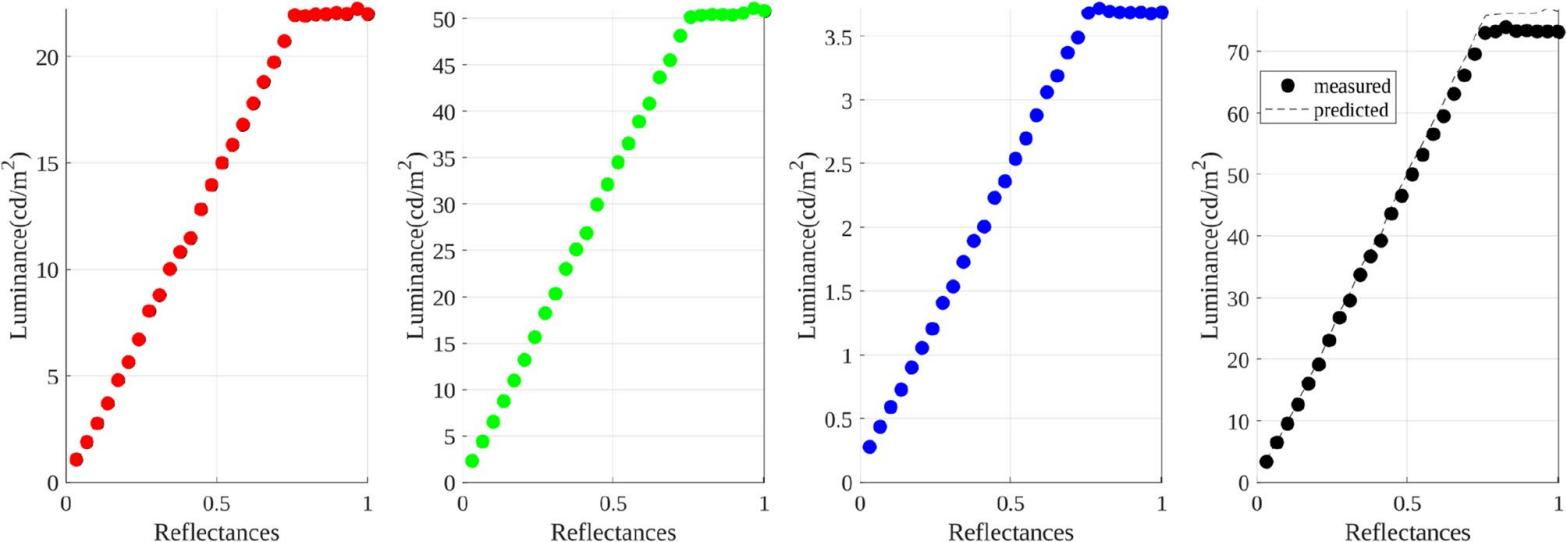
Relationship between input reflectance and luminance values measured with the colorimeter. HMD was controlled via Unreal Engine (tone mapping disabled)

Unreal Engine may be applying post-processing steps to the physically based rendering, e.g., a tone mapping operator (TMO) to convert a high dynamic range scene into a low dynamic one. The application of a TMO leads to difficulties in modelling the curves applied in each colour channel and how it affects and alters the presented colours; for more details on disabling the TMO, please refer to Toscani et al. [30]. Therefore, we turn off the TMO of the engine. As expected, by removing the TMO, a linear relation is obtained between the physically based rendering reflectance values from Unreal, and its displayed luminance output, see Fig. 4. While the scene might appear brighter and light sources might be clipped, we obtain a realistic scene where colours remain consistent.

The work of Toscani et al. [30] described a procedure to define the relation between the reflectance and the *XYZ* values measured by the spectroradiometer. Such a procedure allows us to transform *RGB* values introduced in the engine into predicted *XYZ* (called *nominal values*), and vice versa. Note that the spectroradiometer is always our reference.

At this stage, we must verify that the response of the display is linear under engine control and that chromaticities do not change when measured with different instruments. Hence, we proceed as follows: for each colour channel separately, and for the three channels together (from black $RGB = (0, 0, 0)$ to white $RGB = (1, 1, 1)$), we take 29 measurements, sampling the whole 8-bit range in equal intervals. A total of 116 measurements (*x*, *y*, *Y*) were taken with the imaging colorimeter; see Fig. 4, reflectance versus luminance values *Y*. For each colour channel, we define the following piecewise linear function:

1 $$\begin{aligned} L(r) = {\left\{ \begin{array}{ll} s\times r, &{} \quad s\times r < p\\ p, &{} \quad s\times r \ge p, \end{array}\right. } \end{aligned}$$

where *L* is luminance, *r* the reflectance value, and *s*, *p*, respectively, represent the slope and the clipping value estimated from Fig. 4. Note that if we compare the slope of these results with the ones obtained with CS2000 [30], we get the following relation:

2 $$\begin{aligned} \displaystyle \frac{s_\mathrm{ref}^R}{s_\mathrm{tgt}^R} \approx \frac{s_\mathrm{ref}^G}{s_\mathrm{tgt}^G} \approx \frac{s_\mathrm{ref}^B}{s_\mathrm{tgt}^B}, \end{aligned}$$

where $\mathrm{ref}$ stands for the reference instrument (CS2000), and *tgt* refers to the target instrument (I29). The equivalence of these ratios shows that the chromaticities do not change across instrument measurements.

As mentioned in the previous section, the imaging colorimeter is controlled via Matlab. We connect Unreal Engine and Matlab adopting a transmission control protocol (TCP) to trigger the measurements.

#### Measurement Validation

**Fig. 5:**
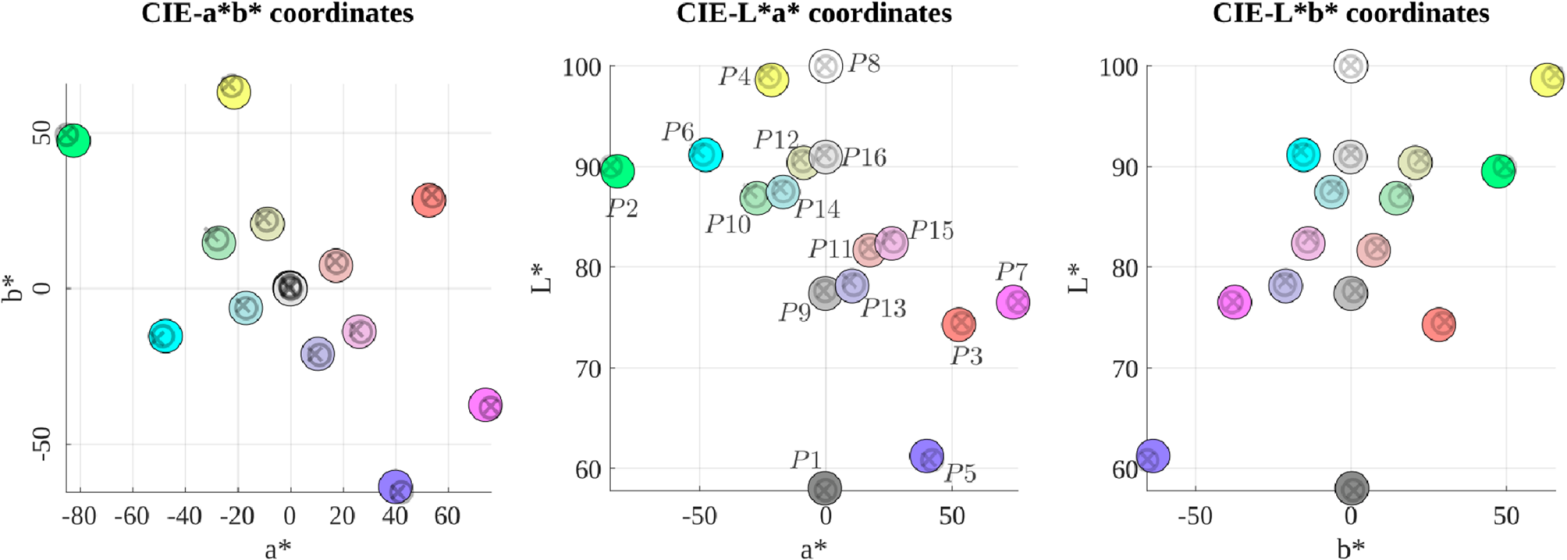
A mixture of colours measured with the spectroradiometer (black circles), measured colours using the colorimeter (black crosses), and the nominal values (colour filled circles). Represented in CIEL*a*b*. HMD was controlled via Unreal Engine (tone mapping disabled)

Again, we display 16 colours spanning the HMD gamut and measure the output with the I29. We verify that the measured values match the nominal values and CS2000 measurements. Figure 5 shows 16 different chromaticities represented in CIEL*a*b* colour space. The 3 subplots, respectively, represent each 2D plane (a*–b*, L*–a*, and L*–b*). The filled coloured circles correspond to the nominal values, the black circles to CS2000, and the black crosses to I29 measurements.

Table 1 reports the colour differences between nominal values versus C2000 and I29 measurements. We compute the $\Delta E_{00}$ metric using Sharma et al. [28] implementation, in CIEL*a*b* colour space. $\Delta E_{00}$ specifies the perceptual difference between two colours, so that values smaller than 2 can be considered as below the threshold of perceptually noticeable difference; see Sharma [27]. These results suggest that the colour differences are not noticeable, and therefore implying that nominal values provided by C2000 and I29 measurements are perceptually equivalent.

**Table 1:** $\Delta E_{00}$ between the nominal values versus CS2000, and I29 measurements. The points correspond to the 16 mixed colours represented in Fig. 5 (CIEL*a*b*)

		P1	P2	P3	P4	P5	P6	P7	P8	P9	P10	P11	P12	P13	P14	P15	P16
$\Delta E_{00}$	CS2000	0.65	0.70	0.57	0.59	0.796	0.34	0.45	0.06	0.96	0.632	0.66	0.58	0.46	0.08	0.36	0.40
	I29	0.71	0.78	0.82	1.04	0.57	1.07	0.46	0.68	1.68	1.68	1.05	1.27	1.06	1.11	0.71	0.90

## Colour Constancy Experiment

**Fig. 6:**
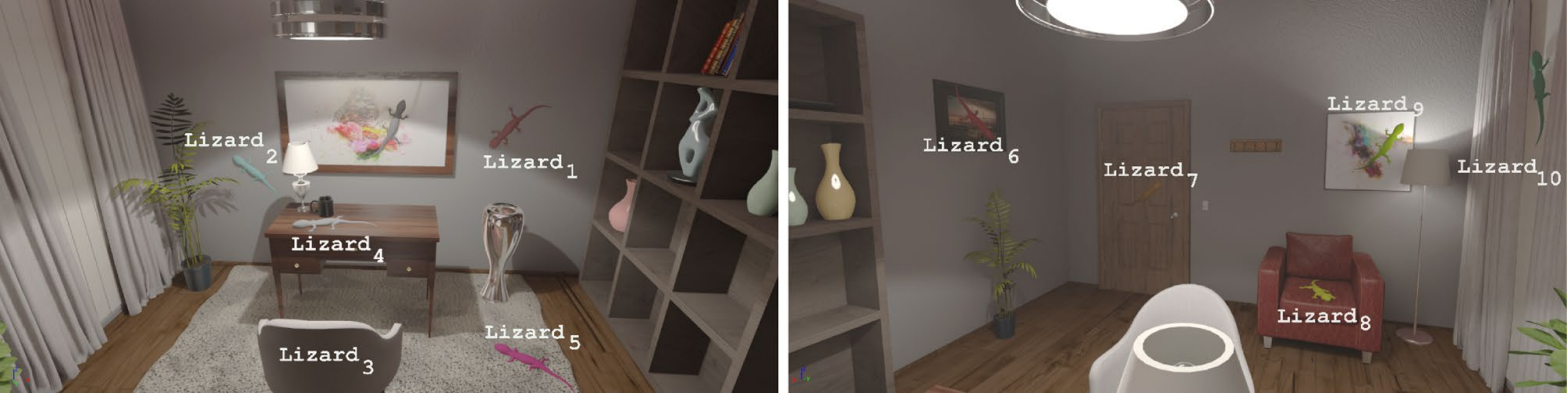
Virtual Reality indoor scene. The room office contains all kind of objects, and within it we select 10 different locations for the object of interest, in this case a *lizard*

The experimental setup consists of an indoor scene (office environment) rendered by the Unreal Engine 4.23. The office room contains matte and glossy objects, and two light sources: one on the ceiling, and a dimmer one at the back of the room; see Fig. 6. Participants were asked to select an achromatic object, in our case a *lizard* that can be placed in 10 distinct positions, within 5 different illuminations (neutral, yellow, blue, green, and red). The illuminants are chosen following Aston et al. [3]: the blue and yellow illuminants lie along the daylight locus, and the green and red along the locus of chromaticities orthogonal in a perceptually uniform chromaticity plane (a*–b* plane in CIEL*a*b*) to the daylight locus at D65 (neutral illuminant). The experiment is defined as follows:

1. the neutral object (grey lizard) is shown under a neutral illumination;
2. then, the office scene is rendered under a different coloured illuminant and there is 1 min of adaptation;
3. for each trial (15 in total), the subject is required to choose one lizard, among 5 placed randomly all over the room and rendered with different reflectances as defined in the RGB colour space. Subjects are instructed to select the one whose colour matches that of the achromatic lizard presented in step 1).

These three steps are repeated for each of the five illuminants.

We adapt the method from Radonjić et al. [25] to our colour constancy experiment. Please note that we only consider the selection task experiment in which the object of interest appears grey under neutral illuminant. First of all, under each illumination (neutral, yellow, blue, green, and red), we compute 10 different colour competitors in CIEL*a*b*: the tristimulus (*T*) match corresponds to a reflectance chosen to produce the same pixel chromaticity as the grey lizard under neutral light (corresponds to $0\%$ colour constant); the reflectance (*R*) match has the same reflectance spectrum as the original grey lizard (corresponds to $100\%$ colour constant); then, six samples equally distributed between *T* and *R*, and finally, two over-constant colours computed from *R*. These are the 10 competitors that would appear randomly in 5 different locations in step 3) above.

To estimate the perceptual achromatic setting for computing colour constancy, we first define a list with all the possible pair comparisons between competitors (a total of 45). Then, for each pair, we count how many times the subjects selected the first element over the second one. We achieve that by considering the subjects’ selection over each of the rest of the competitors present in each trial. After that, we use the method described in Radonjić et al. [25] to estimate the positions of the 10 competitors in a perceptual one-dimensional space, as well as the so- called *selected target position* (i.e., the participants’ ideal colour constant value), following a maximum-likelihood difference scaling optimisation [19]. Notice that the order of the competitors in the estimated subjects’ perceptual domain is maintained as in the CIEL*a*b colour space. From the perceptual domain, we could compute the selected target position in CIEL*a*b* colour space, by preserving the same ratios as in the perceptual domain. Finally, we compute the CCI in CIEL*a*b* as

3 $$\begin{aligned} \displaystyle \mathrm{CCI} = \frac{d(R_\mathrm{neutral}, \mathrm{TP}_\mathrm{colour})}{d(R_\mathrm{neutral}, R_\mathrm{colour})}, \end{aligned}$$

where $d(\cdot , \cdot )$ denotes the Euclidean distance, $R_\mathrm{neutral}$ corresponds to perfect colour constancy value under the neutral illuminant, TP$_\mathrm{colour}$ refers to the selected target position under specific illumination (colour $\in \{$yellow, blue, green, red$\}$), and $R_\mathrm{colour}$ is the perfect colour constancy value under the coloured illuminant.

A total of 11 participants took part in the experiment in two different sessions. To compute the colour constancy index (CCI), we consider two distinct approaches: (1) using participants’ selection colour and its corresponding nominal value, and (2) using participants’ selection and its corresponding imaging colorimeter measurement. We would expect outcomes comparable to Gegenfurtner et al. [15] from both CCIs definitions.

### CCI from Nominal Values

*Nominal* values are the estimated colours from the VR HMD visualisation, i.e., the stimuli presented in the HMD display determined by its reflectance input. The CCI is computed based on the method presented at the beginning of the current section. The main idea of the algorithm is to estimate the selected target positions in the perceptual domain and extrapolated to CIEL*a*b* colour space from the Lab nominal values, following the same relation and ratios as in the perceptual domain. Then, the final CCI per illuminant is calculated from Eq. 3. Please note that, in this case, all the terms in the equation refer to the nominal values.

### CCI from Colorimeter Measurements

The imaging colorimeter allows us to measure exactly the chromaticities presented in the HMD. We proceed in two different ways:

(1) *Colorimeter* We follow again [25] method, and then extrapolate to CIEL*a*b* colour space based on the Lab values of the colorimeter measurements. The CCI is computed following Eq. 3;

(2) *Colorimeter CIELAB* We compute the CCI directly from participants’ selection, i.e., same as in Eq. 3, but instead of considering the estimated target position TP$_\mathrm{colour}$, we replace it with the Lab value of the participants selection. Since participants performed 15 trials per illuminant, we compute the mean of all CCIs per illuminant. Notice that in the latter CCI computation, all the values we refer to are measured with the imaging colorimeter.

**Table 2:** Colour constancy index

	$I_{\text {yellow}}$	$I_{\text {blue}}$	$I_{\text {green}}$	$I_{\text {red}}$
Nominal	0.9005 ± 0.0918	0.8649 ± 0.1440	0.9250 ± 0.0555	0.8879 ± 0.0864
Colorimeter	0.9088 ± 0.0908	0.8787 ± 0.1227	0.9392 ± 0.0402	0.9199 ± 0.0554
Colorimeter CIELAB	0.8733 ± 0.0537	0.8548 ± 0.0841	0.8973 ± 0.0251	0.8976 ± 0.0226

Under each illumination (yellow, blue, green, and red), the mean and standard deviation are presented. Each row represents the CCI computed from (1) the nominal values and method [25], (2) colorimeter measurements and adapting methods from reference [25], and (3) distances in CIEL*a*b* from the colorimeter measurements

Let us describe in detail the process on how to acquire the measurements from participants’ selections. We recorded all tracking data from each subject during the experiment, in particular, HMD position and orientation, along with the lizard position and reflectance at each trial. This provides us with the necessary information to display offline, for each participant and trial, the exact scene they were looking at when selecting the lizard. As mentioned earlier, the I29 is controlled by Matlab via Prometric libraries, and Unreal Engine and Matlab are connected through TCP. Then, for each trial, Unreal displays the exact same scene and I29 captures an image of the display, $I_{xyY}$. To just consider the object of interest, Unreal displays a second scene which masks only the lizard, and we obtain another image from I29, $I_\mathrm{mask}$. We apply an erosion operation to this binary mask, to avoid considering pixels at the edges of the lizard. Finally, the mean is computed from $I_{xyY}$, only on those masked pixels from the eroded mask image: $\displaystyle \mu ( I_{xyY} \cap (I_\mathrm{mask}\ominus s) )$, where $\cap$ denotes intersection, $\ominus$ erosion operation, and *s* the structuring element, in our case a $25\times 25$ square.

### Comparison Between Nominal Values and Colorimetric Measurements

Table 2 presents the CCI values for Sections “CCI from Nominal Values” (first row) and “CCI from Colorimeter Measurements” (second and third row). On one hand, if we focus on the mean, the results show a notable parallel between nominal values and imaging colorimeter measurements. This fact demonstrates the equivalence of both approaches and validates the imaging colorimeter measurements in our colour constancy experiment. On the other hand, there is a slight difference in terms of standard deviation. The reason for this variation might be due to changes in the chromaticity of the lizards in different positions in the room, e.g., dim areas, direct light, or mutual reflections, when taken measurements with the imaging colorimeter.

**Fig. 7:**
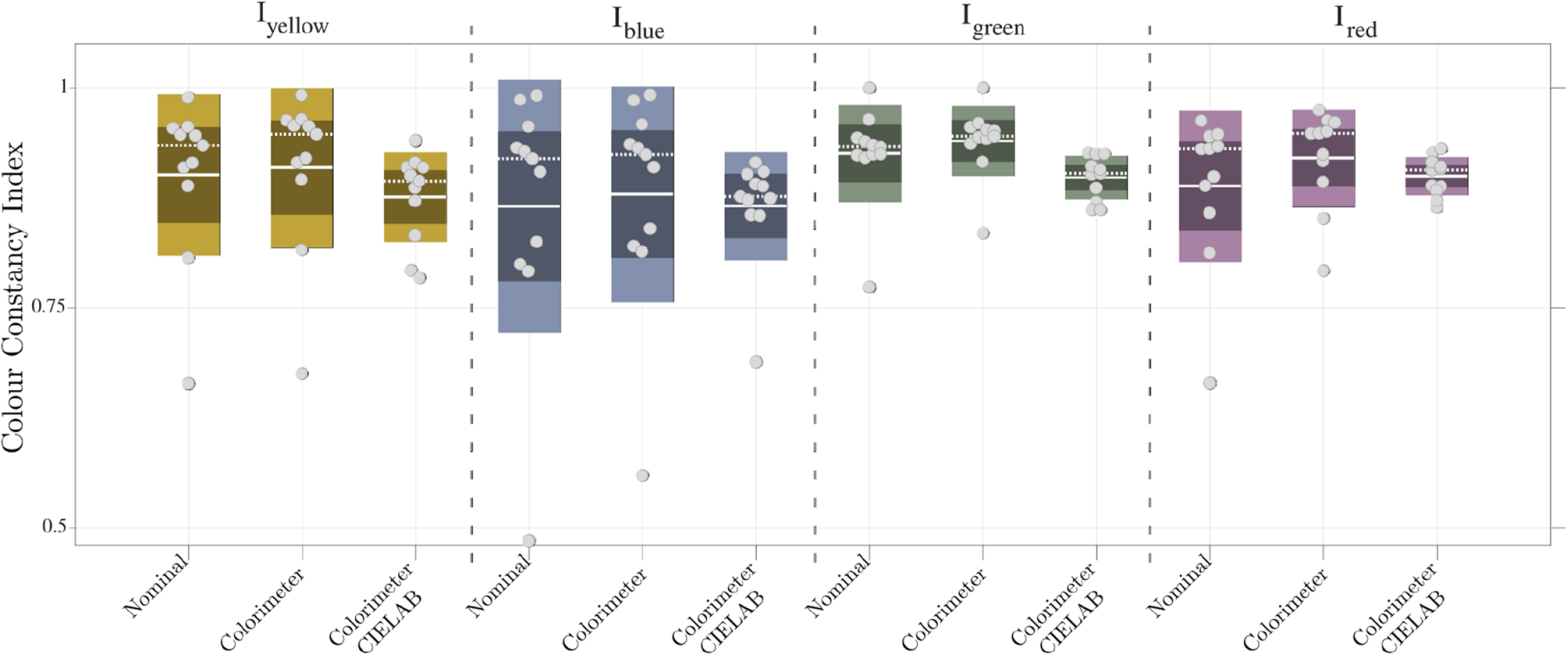
Colour Constancy Index per illuminant. Under each illumination, 3 approaches are presented: *Nominal*, the nominal values and method [25]; *Colorimeter*, the results from colorimeter measurements and adapting method [25]; and *Colorimeter CIELAB*, the mean results from distances in CIEL*a*b* from colorimeter measurements. The circles represent each participant, the continuous lines the mean, the dashed line the median, together with the percentiles

In Fig. 7, we illustrate all the participants’ data (grey circles) per illuminant (yellow, blue, green, and red) with their overall mean (continuous lines), median (dashed lines), and the standard deviation and confidence interval of $95\%$. The plot shows the slight differences within the same lighting conditions. We find a more significant variation under the red and green illuminations, and minor in yellow and blue. These differences are more evident in *Colorimeter CIELAB* approach, where the data variability is reduced by at least $40\%$.

### Comparison with Previous Findings

In our experiment, colour constancy ranged between $85\%$ and $94\%$, similar to previous colour constancy experiments in real-world scenes [15, 18], where authors reported up to $80\%$ and above $90\%$ constancy, respectively. Constancy has often been found to be best for blue daylight illuminants (e.g., [12], [21]). However, our results follow the previous reports from experiments with natural scenes [7, 34], in which there are no significant differences in colour constancy between illumination conditions. A simple explanation for this discrepancy could be that the high level of constancy performance hides the differences between the illumination conditions (i.e., ceiling effect). The agreement of our results with the previous research in natural conditions corroborates the validity of the proposed VR setup.

## Conclusion

We presented a framework to colour validate an HMD calibration for VR using the imaging colorimeter I29. As we have shown, when no rendering software is involved in the visualisation pipeline, the HMD behaves as a conventional monitor. Therefore, we could calibrate it based on standard procedure [8]. In this scenario, the HMD follows a power-law function (gamma curve of approximately 2.2). On the contrary, when a software rendering is considered, e.g., Unreal Engine 4.23, the presented stimuli are troublesome to model, as already shown in Toscani et al. [30]. Among other factors, the additional complexity in modelling the HMD response using the game engine is due to the default application of post-processing steps, such as colour correction and tone mapping curve. Consequently, we disable all post-processing stages including the tone mapping operator. This allows us to calibrate the HMD as previously done for the simpler scenario (HMD as a monitor), and we found a linear relationship between input reflectances and presented stimuli, while reaching saturation at specific values for each channel. We only considered matte surfaces for the rendering calibration.

Moreover, we studied and verified the described colour calibration within a colour constancy experiment Gil Rodríguez et al. [16]. This experiment presents a selection task paradigm of an achromatic object within 5 different illuminations. We measured with the imaging colorimeter all the participants’ choices. In this way, we could compare the CCI results obtained from [16], using the nominal values, and the ones measured directly with the colorimeter. Overall, we demonstrated that the chromaticity of the stimuli presented in the HMD can be controlled based on the previous characterisation, in a colour constancy experiment. Furthermore, we showed that colour constancy performance in an immersive realistic VR environment is similar to what is reported for natural scenes, e.g., (Kraft and Brainard [18]).

Since game engines, such as Unreal Engine, allow us to change surround and lighting conditions in a controlled and simple manner, future research will be aimed at determining the influence of different cues in colour constancy, including for instance local surround of the object and specular highlights in glossy objects. Providing participants with the possibility to interact with the scene, by directly manipulating the displayed object, would also represent an interesting venue for additional research.

## Footnotes

1 In the configuration of the four-colour calibration, the *distortion calibration* was set to *none*. For the rest of measurements, it was set to *ARVR Lens 024@0.3m.*
